# The impact of a dedicated checklist on the quality of onsite management of critically buried avalanche victims in cardiac arrest in a Swiss helicopter emergency medical service

**DOI:** 10.1186/s13049-024-01300-3

**Published:** 2024-12-03

**Authors:** Maxime Trolliet, Mathieu Pasquier, Marc Blancher, Roland Albrecht, Alban Lovis, Hermann Brugger, Alexandre Kottmann

**Affiliations:** 1 Department of Anesthesiology, EHNV, Hospital of Yverdon, Rue d’Entremonts 11, Yverdon-les-Bains, 1400 Switzerland; 2 Department of Emergency Medicine, Lausanne University Hospital and University of Lausanne, CHUV, Rue du Bugnon 46, Lausanne, 1011 Switzerland; 3 Emergency Department, University Hospital of Grenoble-Alpes and French Mountain Rescue Association ANMSM, Grenoble Cedex 09, 38043 France; 4 Swiss Air Ambulance, Rega, P.O. Box 1414, Zurich, 8058 Switzerland; 5 Department or Emergency Medicine, Bern University Hospital, Inselspital, University of Bern, Bern, 3010 Switzerland; 6 Division of Pulmonary Medicine, Lausanne University Hospital and University of Lausanne, CHUV, Rue du Bugnon 46, Lausanne, 1011 Switzerland; 7 Institute of Mountain Emergency Medicine, Eurac Research, Via Ipazia 2, Bolzano, 39100 Italy

**Keywords:** Avalanche, Cardiac arrest, Checklist, Quality, Compliance, Documentation

## Abstract

**Background:**

The management of avalanche victims in cardiac arrest (CA) is a challenging situation for rescuers. Despite existing specific management algorithms, previous studies have reported poor compliance with international guidelines and incomplete documentation and transmission of the information required for patient management. The Avalanche Victim Resuscitation Checklist (AVRC) was developed in 2014 in response by the International Commission for Mountain Emergency Medicine. Our aim was to assess the impact of the AVRC on the quality of onsite management of critically buried avalanche victims in CA, i.e. the compliance of management with international guidelines and the completeness of documentation of avalanche specific information.

**Methods:**

We assessed compliance and documentation in a Swiss helicopter emergency medical service (HEMS) between January 2010 and April 2020. Victims buried for more than 24 h were excluded.

**Results:**

In the 10-year study period, 87 critically buried avalanche victims in CA were treated by the HEMS, 44 of them after the introduction of the AVRC. Enough information was available to assess management compliance in over 90% of cases (*n* = 79). Inadequate management (*n* = 25, 32%) and incomplete documentation occurred more often in patients with a long burial duration. After the introduction of the AVRC, the compliance of patient management with the guidelines increased by 36% (from 59 to 95%, *p* < 0.05) and led to complete documentation of the required information for patient management.

**Conclusions:**

The use of the AVRC improves the quality of management of critically buried avalanche victims in CA and ensures complete documentation of avalanche specific information. Quality improvement efforts should focus on the management of avalanche victims with a long burial duration. The use of the AVRC enables identification and appropriate treatment of patients with hypothermic cardiac arrest.

**Supplementary Information:**

The online version contains supplementary material available at 10.1186/s13049-024-01300-3.

## Background

Avalanches claim a significant number of victims each year, most of them dying from asphyxia during critical burial, with some experiencing trauma and/or hypothermia. The pre-hospital management of critically buried avalanche victims in cardiac arrest (CA) should follow international guidelines and the respective algorithms [1–3]. The application of these algorithms requires specific information: burial duration, airway patency and core temperature and CA rhythm (ECG) at extrication [1–5]. Although applying these algorithms seems easy, poor compliance of patient management with the international guidelines has been described [6]. 

The presentation of the preliminary results of the study by Strapazzon et al. to the International Commission for Mountain Emergency Medicine (ICAR MEDCOM) in 2012 was the trigger for the development of the first Avalanche Victim Resuscitation Checklist (AVRC) by the ICAR MEDCOM, established in 2014 and based on the international guidelines for the management of avalanche victims, as presented in the Additional file 1 [6, 7]. 

The aim of the AVRC was to improve compliance with the guidelines by helping rescuers in the decision-making process for these rare and usually complex situations, as well as to improve documentation and data transfer to hospital [1, 2, 7, 8]. 

Our main objective was to assess whether the introduction and use of the AVRC in a Helicopter Emergency Medical Service (HEMS) in Switzerland had an impact on the quality of the onsite management of critically buried avalanche victims in CA.

## Method

We retrospectively analysed the patients caught by an avalanche and rescued by the Swiss HEMS Rega – Swiss Air Ambulance between January 2010 and April 2020. We included all critically buried avalanche victims in CA at extrication. Patients with respiratory arrest only or not critically buried or found more than 24 h after the emergency call were excluded.

Rega is the major HEMS in Switzerland and has 13 bases distributed along the Alps [9]. Every base is equipped with a single helicopter dedicated to HEMS missions and available 24/7. The standard crew includes a pilot, a paramedic and an emergency physician. The AVRC was introduced at Rega at the beginning of the winter season 2014/2015 and was recommended to be used for every avalanche victim. Every helicopter was equipped with 10 AVRCs and all emergency physicians and paramedics completed a standardised e-learning course as recommended by the ICAR MEDCOM [7]. 

Data were extracted from the pre-hospital medical record (PHMR, as presented in the Additional file 2) and, if available, from the AVRC, both completed by the emergency physician for every patient. The following general information was extracted: month and year of the accident, age and sex of the patient, presence of trauma, pre-hospital NACA score (used to grade the severity of the patient’s medical condition) [10], provision of cardiopulmonary resuscitation, no flow and low flow duration (defined as the duration of CA without and with chest compression, respectively), medical treatment and the destination hospital. We also extracted the following avalanche-specific information: safety concerns for the rescue team (based on explicit written information on the PHMR, e.g. the risk of a second avalanche, emergency extrication), burial degree, burial duration, presence of signs of life, airway patency, presence of an air pocket, presence of obvious lethal injuries, initial CA rhythm and core temperature. In the case of contradictory information between the AVRC and the PHMR, the latter was used as it is the official medical document that is used in every case to pass information to hospital staff. All extracted data were transcribed in a database.

The quality of avalanche victim management in CA was assessed by analysing both the compliance of avalanche victim management with international guidelines and the completeness of the documentation of avalanche-specific information on the PHMR and/or, if applicable, on the AVRC.

The compliance of avalanche victim management with the guidelines was assessed independently by two authors (MP, MB) who were blinded as to whether or not the AVRC was used. Consensus was reached on cases with a discrepancy at a consensus meeting moderated by a third expert, at which these cases were reviewed by the 2 experts. Management was considered adequate when it complied with the avalanche victim management guidelines regarding the indication to start or stop resuscitation, the indication to transport the patient to the hospital under resuscitation and the type of hospital of destination (extracorporeal life support [ECLS] centre or not). Management was considered inadequate in every situation in which the care given did not comply with the guidelines, and doubtful when the information available did not allow management compliance to be assessed. The 2001 guidelines were used to assess management compliance up to the winter of 2012–2013, those of 2013 up to the winter of 2014–2015 and those of 2015 for interventions that took place starting from the winter of 2015–2016 [1–3]. Airways that were clearly documented as obstructed were considered as such. As airway obstruction occurring in avalanche accidents is defined by the presence of compacted snow in the nose and mouth, mentions of blood or vomit in the mouth was not considered airway obstruction [3, 11]. In line with the evolution of the international guidelines, a short burial was defined as < 35 min before November 2015 and as < 60 min since then [2, 3, 12]. 

Pre-hospital documentation was considered complete when all information required for management of the avalanche victim – hence the application of the algorithm for the given situation – was identifiable on the PHMR and/or, if applicable, on the AVRC.

The study received approval from the Cantonal Commission for Ethics in Human Research (Zürich, Switzerland) on 6 March 2020 (protocol no. 2020-048).

### Statistical analysis

The data extracted from the PHMRs and AVRCs were transferred into an Excel file by the main investigator (MT), and then coded and analysed by using STATA version 14 software (Stata Corporation, College Station, TX, USA). Quantitative variables were described as mean ± standard deviation or median (interquartile range), depending on their distribution. A Student’s t-test or Mann-Whitney U test were used to compare continuous variables between the different groups. Categorical variables were described according to their number and percentage. They were compared by using a chi-squared test when the number of observations was sufficient (> 5 observations per group) or by Fisher’s exact test otherwise. Cohen’s kappa coefficient was used to measure interrater agreement. A *p*-value of less than 0.05 was used to indicate statistical significance.

## Results

**Fig. 1:**
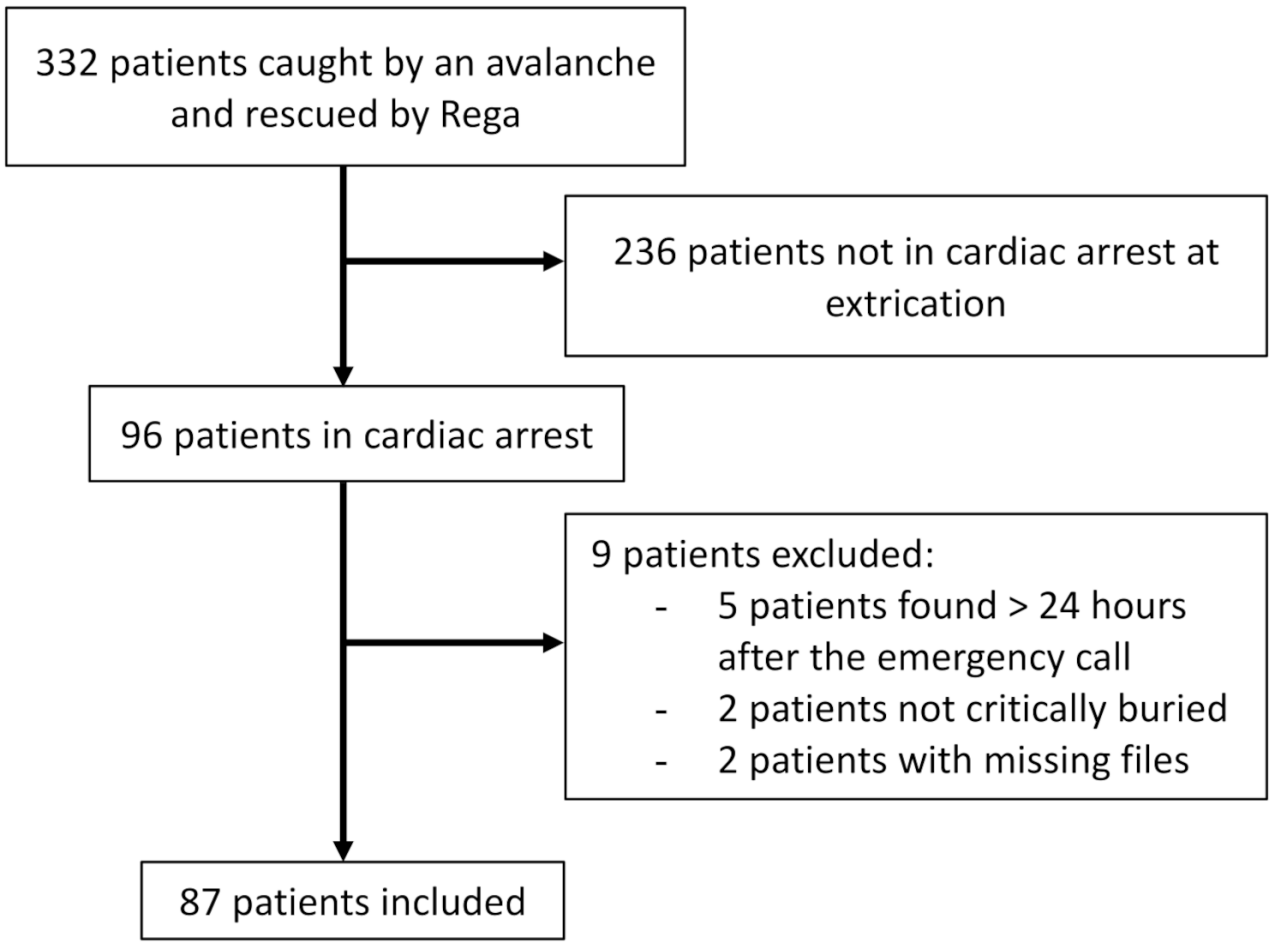
Flowchart of study patients. Patients in cardiac arrest after being critically buried in an avalanche, Rega – Swiss Air Ambulance, Switzerland, between January 2010 and April 2020

Among the 322 avalanche victims rescued by Rega between 2010 and 2020, 96 were found in CA, and, of these, 87 were included in the study (Fig. 1). The patients were mostly male (90%, *n* = 82), with a mean age of 42 ± 13 years (range 7–82 years).

Characteristics of patients in CA and their management are shown in Table 1. Forty-nine (56%) of the 87 included patients were declared dead on scene, 20 of whom (41%) had no resuscitation attempt. Of the 38 (44%) patients transported, 24 (63%) were transported under resuscitation, and 14 (37%) had return of spontaneous circulation onsite.

**Table 1:** Patients in cardiac arrest after being critically buried in an avalanche, Rega – Swiss Air Ambulance, Switzerland, 2010–2020 (*n* = 87)

	Total (*n* = 87)	Transported to hospital (*n* = 38)	Declared dead on scene (*n* = 49)	*p*-value
**Witnessed CA**, ***n*** **(%)**	1 (1)	1 (3)	0	
***No flow duration (minutes), median (IQR) (*** ***n*** ** = 74)**	46 (32–90)	45 (30–67)	60 (38–510)	**< 0.001**
**Low flow duration (minutes), median (IQR) (** ***n*** ** = 79)**	25 (0–45)	40 (17–60)	10 (0–30)	**< 0.001**
**Bystander CPR**, ***n*** **(%)**				**0.660**
No	64 (74)	29 (76)	35 (71)	
Yes, by companions	13 (15)	4 (11)	9 (18)	
Yes, by rescuers	7 (8)	4 (11)	3 (6)	
Not documented	3 (3)	1 (3)	2 (4)	
**CPR duration by HEMS crew (minutes), median (IQR) (** ***n*** ** = 58)** ^**a**^	29.5 (10–65)	25 (10–57) *n* = 21	30 (10–58) *n* = 37	**0.802**
**Intubation**, ***N*** **(%)**	42 (48)	30 (79)	12 (24)	**< 0.001**
**Intraosseous access**, ***n*** **(%)**	23 (26)	16 (42)	7 (14)	**0.004**
**Intravenous access**, ***n*** **(%)**	25 (29)	18 (47)	7 (14)	**0.001**
**Temperature measurement**, ***n*** **(%)**	34 (39)	22 (58)	12 (24)	**0.002**
**Use of multi-functional electrodes**^**b**^, ***n*** **(%)**	26 (30)	20 (53)	6 (12)	**< 0.001**
**Resuscitation on site**, ***n*** **(%)**^**a**^	67 (77)	38 (100)	29 (59)	**< 0.001**
**Mechanical chest compression device**^**c**^, ***n*** **(%)**	18 (33)	15 (54)	3 (11)	**0.001**
**Mechanical chest compression device**^**c**^ **on standby**, ***n*** **(%)**	7 (13)	7 (25)	0	**0.005**
**Defibrillation attempted**, ***n*** **(%)**	10 (11)	7 (18)	3 (6)	**0.074**
**Adrenaline dose (mg), median (IQR) (** ***n*** ** = 86)**	0 (0–2)	1 (0-3.4)	0 (0–0)	**0.010**
**Infusion volume (L of crystalloids), median (IQR; range) (** ***n*** ** = 86)**	0 (0–0;0–1)	0 (0–0;0–1)	0 (0–0;0-0.5)	**0.033**

CA = cardiac arrest; CPR = cardiopulmonary resuscitation; HEMS = helicopter emergency medical service; IQR = interquartile range

^a^CPR duration by HEMS crew only available for 58 of 67 patients resuscitated onsite. Five missing values and four victims resuscitated by bystanders only

^b^Electrodes used for both defibrillation and rhythm check

^c^Introduced in 2013

The specific information required to follow international guidelines for the onsite management of critically buried avalanche victims in CA is shown in Table 2. While burial time was recorded for almost all patients (*n* = 95, 98%), less than half of them (*n* = 41, 47%) had a temperature recorded. However, according to the algorithm, temperature would have been required to guide medical management in only two (2%) of the patients in whom it was not measured. Not documented items are regressing after the introduction of the AVRC even though it was not used on each rescue.

**Table 2:** Specific information required to follow international guidelines for the onsite management of critically buried avalanche victims in cardiac arrest, before and after the introduction of the Avalanche victim resuscitation checklist (AVRC). Patients in cardiac arrest after being critically buried in an avalanche, Rega – Swiss Air Ambulance, Switzerland, 2010–2020 (*n* = 87)

	Total (*n* = 87)	Before the introduction of the AVRC (2010–6.2014) *n* = 43	After the introduction of the AVRC (7.2014–2020) *n* = 44	*p*-value
**Safety concern onsite (*n* = 86)**, ***n* (%)**	**6 (7)**	**4 (10)** ***n* = 42**	**2 (5)** ***n* = 44**	**0.395**
**Frozen body**, ***n* (%)**				**0.115**
No	67 (77)	30 (70)	37 (84)	
Yes	10 (11)	5 (12)	5 (11)	
Not documented	10 (11)	8 (19)	2 (5)	
**Non-compressible thorax**, ***n* (%)**				**0.066**
No	67 (77)	31 (72)	36 (82)	
Yes	5 (6)	1 (2)	4 (9)	
Not documented	15 (17)	11 (26)	4 (9)	
**Lethal injuries**^**a**^, ***n* (%)**				**0.315**
No	76 (87)	36 (84)	40 (91)	
Yes	2 (2)	2 (5)	0	
Not documented	9 (10)	5 (12)	4 (9)	
**Associated trauma**, ***n* (%)**				**0.613**
No	13 (15)	5 (12)	8 (18)	
Yes	33 (38)	18 (42)	15 (34)	
Not documented	41 (47)	20 (47)	21 (48)	
**Burial time (minutes), median (IQR) (** ***n*** ** = 72)**	**45 (30–92)**	**60 (25–660)** *n* = 33	**45 (10–410)** *n* = 39	**0.220**
**Burial time cutoff**^**b**^, ***n* (%)**				**0.035**
Short burial	35 (40)	12 (28)	23 (52)	
Long burial	50 (57)	29 (67)	21 (48)	
Not documented	2 (2)	2 (5)	0	
**Temperature** ^**c**^ **(°C), median (IQR) (** ***n*** ** = 39)**	**30.8 (27.5–32.2)**	**29.6 (22–32)** *n* = 15	**31.3 (28.2–32.8)** *n* = 24	**0.031**
**Temperature cutoff**^**b, c**^, ***n* (%) (*n* = 41)**				**0.029**
< 32/<30 °C	24 (28)	11 (26)	13 (30)	
≥ 32/≥30 °C	17 (20)	4 (9)	3 (30)	
Not documented	46 (53)	28 (65)	18 (41)	
**Airway**, ***n* (%)**				**0.255**
Obstructed airway^d^	26 (30)	13 (30)	13 (30)	
Free airway	44 (51)	19 (44)	25 (57)	
Documented unknown	1 (1)	0	1 (2)	
Not documented	16 (18)	11 (26)	5 (11)	
**Air pocket**, ***n* (%)**				**0.484**
No	20 (23)	8 (19)	12 (27)	
Yes	27 (31)	13 (30)	14 (32)	
Documented unknown	8 (9)	3 (7)	5 (11)	
Not documented	32 (37)	19 (44)	13 (30)	
**Initial cardiac rhythm**^**e**^, ***n* (%)**				**0.195**
Asystole	58 (67)	28 (65)	30 (68)	
Pulseless electrical activity	5 (6)	2 (5)	3 (7)	
Ventricular fibrillation	3 (3)	0	3 (7)	
Documented unknown	1 (1)	0	1 (2)	
Not documented	20 (23)	13 (30)	7 (16)	

IQR = interquartile range

^a^Decapitated patient or transection of the trunk only

^b^Cutoff change in November 2015 with the publication of the European Resuscitation Council guidelines in 2015 by Truhlar et al. [3] The burial duration cutoff value to differentiate a short from a long burial duration changed from ≤ 35 min to ≤ 60 min, and the cutoff value for core temperature changed from < 32 °C to < 30 °C. In the case of divergence between the management indicated by the burial time and that indicated by the temperature of the same patient, the appropriate management was considered to be that indicated by the temperature value. Two patients had a cutoff value without any temperature measurement. The first had a 10-minute burial (therefore hypothermia < 30 °C excluded). The second had a normal level of consciousness after a return of spontaneous circulation (ROSC) (therefore hypothermia < 30 °C excluded by clinical means)

^c^Core temperature were measured by oesophageal probe

^d^Compacted snow in the nose and mouth or if described as “obstructed” by the physician

^e^One of the 87 patients (1.1%) developed cardiac arrest (rescue collapse) during management after reaching ROSC and was further transported to hospital under resuscitation

Management compliance could not be assessed in 8 (9%) of the 87 cases due to lack of information, but enough information was available for the other 79 (91%) cases. In 56 (71%) of them, the two experts had an identical opinion on compliance (Kappa = 0.4037). In the other 23 cases (29%), they had different opinions: 12 (15%) cases with opposite opinions (adequate vs. inadequate) and 11 cases in which one of the experts expressed a doubt. A consensus was found for these 23 cases with an initial discrepancy. Management was considered adequate in 54 of these 79 cases (68%) and inadequate in 25 (32%). Inadequate management occurred more often in patients with a long burial duration (*n* = 19) than in patients with a short burial duration (*n* = 6) (76% vs. 24%, respectively, *p* < 0.001). The main deviations from the guidelines identified for the onsite management of avalanche victims in CA are given in Table 3. Overall, the documentation was complete in 72 cases (83%), and 13 (87%) of the 15 cases with incomplete documentation had a long burial duration (burial duration was missing for the two remaining patients).

**Table 3:** Details of the 25 cases, for which management was considered inadequate. Patients in cardiac arrest after being critically buried in an avalanche, Rega – Swiss Air Ambulance, Switzerland, 2010–2020 (*n* = 87)

	*n* (%)
**Over-treatment**	**10 (40)**
Transport with ongoing CPR while not appropriate	0
Too long resuscitation duration	9 (36)
Resuscitation started while not indicated	1 (4)
**Under-treatment**	**15 (60)**
No resuscitation initiated while indicated	5 (20)
Premature resuscitation termination	5 (20)
Transport to non-ECLS centre while ECLS centre orientation recommended	5 (20)

CPR = cardiopulmonary resuscitation; ECLS = extracorporeal life support

**Fig. 2:**
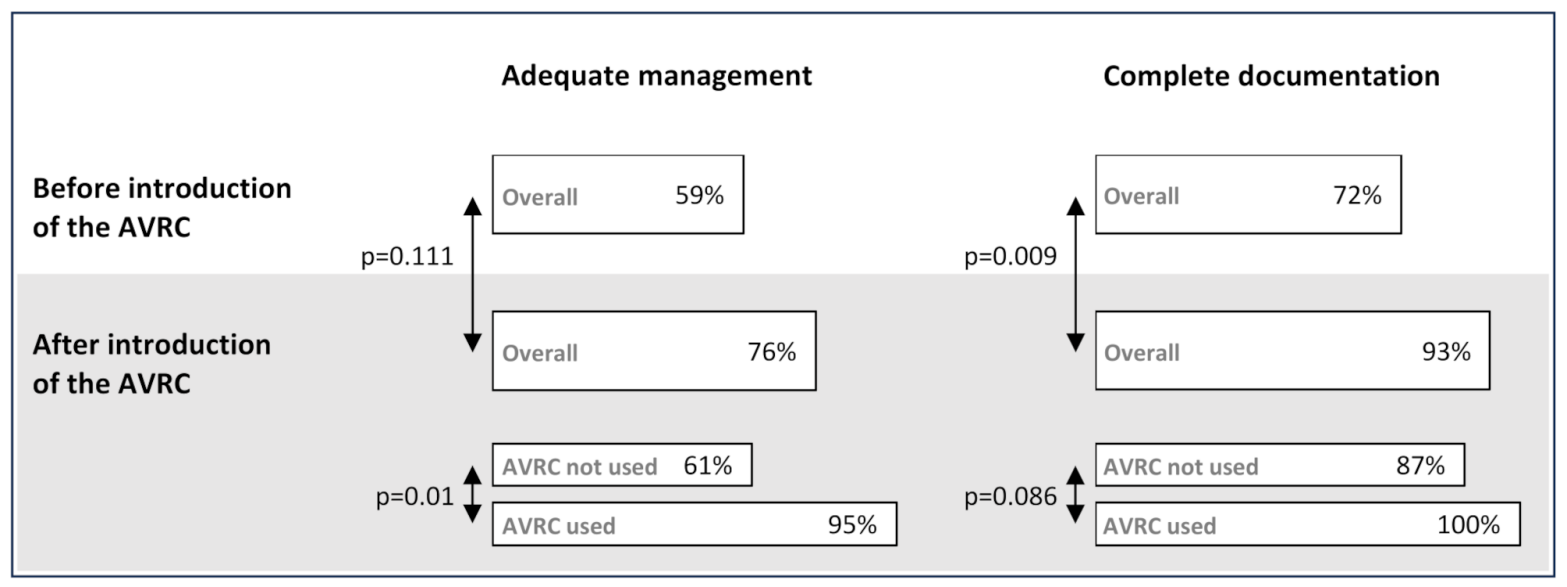
Impact of the introduction and use of the Avalanche Victim Resuscitation Checklist (AVRC) on the rates of adequate management of critically buried avalanche victims in cardiac arrest and of complete documentation of avalanche-specific information. Patients in cardiac arrest after being critically buried in an avalanche, Rega – Swiss Air Ambulance, Switzerland, 2010–2020 (*n* = 87)

After its introduction, the AVRC was used in 21 (48%) of the 44 patients. The impact of the introduction of the AVRC on management quality is illustrated in Fig. 2. Overall compliance with international guidelines did not increase significantly with the introduction of the AVRC (76% after introduction vs. 59% before, *p* = 0.111), but management compliance was significantly higher when the AVRC was used than when it was not (95% vs. 61%, respectively, *p* = 0.01). The rate of documentation completeness increased after the introduction of the AVRC in general (93% after introduction vs. 72% before, *p* = 0.009), but there was no significant difference between the group who used the AVRC and the group who did not (100% vs. 87%, respectively, *p* = 0.086).

## Discussion

Compliance of onsite management of avalanche victims with international guidelines has been addressed in two studies [6, 13]. The present study is, however, the first to assess the impact of the AVRC on the quality of pre-hospital management of avalanche victims in CA. The use of the AVRC was associated with an increase in the quality of management of critically buried avalanche victims in CA, as defined by the rates of adequate management and complete documentation [2, 3, 12]. 

## Compliance of victim management with guidelines and judgement criteria

The rate of adequate management in our study is similar to that reported by Metrailler-Mermoud et al. (68% vs. 71%, respectively), although the rates of inadequate management are different (32% vs. 14%, respectively). In both studies, under-treatment (19% and 11%, respectively) was found more frequently than over-treatment (13% and 3%, respectively) [13]. Strapazzon et al. also report that inadequate management is common, both in terms of over- and under-treatment [6]. 

These differences between studies could be explained by the additional judgement criteria (duration of resuscitation and type of destination hospital) that were assessed in our study, but not in the study by Metrailler-Mermoud et al. [13]. For instance, in the latter, the question of the appropriate hospital destination did not arise, as all patients were transported to either the regional referral hospital or the nearest university hospital, both of them being ECLS centres [13]. 

Just as there may be good reasons to continue resuscitation beyond the 20 min recommended by the guidelines for normothermic patients in CA, the transport of a hypothermic patient in CA to a non-ECLS hospital when an ECLS hospital would have been indicated can also be justified. These decisions are made on the basis of the clinical and operational context specific to avalanche rescue and must also take into account the available resources, in this case almost exclusively HEMS. Indeed, avalanche accidents involving several critically buried people, or even several people in CA, are relatively frequent (14% and 5%, respectively) [8]. Moreover, avalanche accidents are often concentrated on so-called high-risk days, linked to avalanche risk, weather conditions and the practice of snow sports outside of safe ski areas [14]. Since the indication for ECLS rewarming with the hypothermia outcome prediction after ECLS (HOPE) score can be made in a non-ECLS hospital, direct transport to an ECLS centre might be reserved for patients for whom it is truly indicated on days when resources are limited [15, 16]. 

The assessment of management compliance through judgment criteria is based on the available documented information from the observation and interpretation of clinical parameters. For instance, Metrailler-Mermoud et al. used an interpretation of airway patency other than that used in our study [13]. Airway patency might be more open to interpretation than other parameters are, such as ECG or temperature, but is especially key in decision-making for the management of avalanche victims with a long burial, as a patent airway maximises chances of survival [17]. Acknowledging this, and in order to reduce misinterpretation, a clear definition of airway patency is featured in the latest ICAR MEDCOM recommendations [4]. 

**Where to focus: most frequent situations with inadequate management**.

Both in our study and that of Metrailler-Mermoud et al., decision-making assessed as inadequate was predominantly committed in patients with a long burial duration (76% and ≥ 67%, respectively) [13]. Strapazzon et al. also reported resuscitation of only 51% (*n* = 24) for victims with a long burial duration and a patent or undetermined airway [6]. 

There are several possible reasons for this finding. First, managing patients in this category is more complex, as more information needs to be gathered to apply the algorithm [1–5, 18]. Second, the management of these patients is specific to avalanche accidents and cannot be based on experience. In fact, it is difficult to gain much experience, as avalanche accidents remain rare for HEMS rescuers and long burial situations have become even less frequent in our setting since the burial time cutoff value was moved to 60 min in 2015 [3, 8]. 

Finally, rescuers (especially non-medical personnel) might wrongly compare the prognosis of patients with a long burial (i.e. with potential hypothermic CA) to that of patients with a short burial (mostly hypoxic, normothermic CA) or with other causes of CA [11, 16]. This can lead to fatalistic thoughts (e.g. “anyway, after so much time under the snow, he is dead”) and thus arbitrary and inadequate decision-making, when in fact the prognoses of hypothermic and normothermic CA are completely different, as are the indications to pursue advanced resuscitation efforts (including ECLS) [19]. Indeed, among the victims with a long burial duration are cases (albeit infrequent) of those in hypothermic CA (i.e. having hypothermia as a reversible cause of CA, without prior hypoxia), who may have a good chance of survival without neurological sequelae after ECLS rewarming [4, 11, 17–22]. 

Therefore, quality improvement efforts should focus on the management of avalanche victims with a long burial duration. Patients must be carefully assessed and the specific information thoroughly collected and documented. Each step of the algorithm must be checked to absolutely avoid under-treatment of a patient with a potentially good outcome. This is exactly the purpose of a checklist, defined as a tool designed to ensure that a procedure is performed as planned by checking that all of the important preparations have been completed beforehand [23]. 

## Quality improvement using the avalanche victim resuscitation checklist

We were able to demonstrate that the use of the AVRC was associated with an increased rate of adequate management of critically buried avalanche victims in CA from around 60–95%. This finding reinforces the results of previous studies that have shown that checklists are an efficient intervention to improve processes of care such as the application of guidelines [24–28]. Unlike other checklists that are sometimes a simple set of checks, the AVRC was deliberately conceived as a tool based on and including an algorithm, serving as clinical decision support [27]. This is an important feature of the AVRC, as stressful situations such as avalanche accidents might impair memory function, and cognitive aids such as checklists have been shown to improve performance in healthcare for solving complex and time-critical tasks [29–31]. 

Over the entire study period, documentation completeness was significantly higher when the checklist was used than when it was not (100% vs. 77%, respectively, *p* = 0.016). The AVRC was also designed to document the information required for the management of avalanche victims in CA both pre-hospital and in-hospital [15]. It is crucial that this information accompany the patient to the hospital and is directly available. Although reviewing the PHMRs makes it possible to find this information most of the time, this work can be tedious, whereas it is directly available on the AVRC, confirming that checklists can lead to improved information flow [32]. In contrast to the PHMRs, the AVRC offers the possibility to document information in a categorical way as well (e.g. burial duration “>35/60 minutes” or “≤35/60 minutes”), allowing documentation even when it is not precisely known. Although categorisation is less precise than with the use of a continuous variable, it is the cutoff that is relevant in this case and allows progress in the algorithm. The same is true for airway patency, ECG and core temperature, as the AVRC is non-blocking, allowing providers to progress through the algorithm although some information might not be available.

Finally, the impact of a checklist, as with any clinical intervention, depends on its correct use and on the compliance of the providers who use it [33]. We found an overall rate of adequate management of only 76% after the introduction of the AVRC, largely influenced by its low usage rate of 48%. Compliance and barriers to implementation are recurrent challenges in quality improvement programs and efforts will focus on increasing compliance and perpetuating the use of the AVRC in our service [34]. The AVRC was recently updated by the ICAR MEDCOM according to the latest European Resuscitation Council guidelines, confirming the added value of its use in rescue operations. The results of this study, together with the new version of the AVRC, which is identical to the algorithm and more faithful to the chronological stages of management in the field, should increase acceptance, thereby boosting compliance and facilitating implementation in peer services [4, 5]. 

### Limitations

The main limitations of this study are its retrospective design and the small sample size. Avalanches accidents, moreover involving victims in cardiac arrest, are rare events, implicating a small sample size despite an extended study period. However, this might be outweighed by the strengths of the study being conducted in a single HEMS with uniform SOPs & trainings and one of the highest exposure to avalanche accidents amongst HEMS worldwide. Eight cases were excluded because not enough information were available to draw an objective conclusion on the management adequacy. However, including these patients would have influenced the results concerning documentation completeness, and potentially also those concerning management adequacy, in favor of the checklist.

Another limitation can be seen in the discrepancy between the two assessors. Although this can be interpreted as the subjective nature of expert opinion at first glance, it actually more depicts the difficulty to assess the adequacy to algorithms and to establish rules to do so. Discrepancy between the two experts concerned almost exclusively (91%, *n* = 21) the decision to start or stop resuscitation and in particular the duration of resuscitation before stopping. For example, in a patient with a short burial time, resuscitation should last 20 min before being stopped, unless otherwise specified. Similarly, in a patient with a long burial time and an obstructed airway, resuscitation should be started before being stopped once asystole has been confirmed. Although these rules were explicitly described in the expert instructions, they seem to have been forgotten by the experts and might explain why one of the experts had a doubt about compliance for nearly half of the cases with discrepancy. Further, once these rules have been reminded at the start of the consensus meeting, no discussion was needed for most cases, as both experts immediately agreed.

In addition, some of the authors were involved in the development and update of the AVRC. However, the assessors were blinded to the use of the checklist. Finally, our study is based only on pre-hospital data collection. The absence of information on hospital management does not allow us to draw conclusions about the potential impact of the use of the AVRC on patient outcomes. This was, however, not the objective of the study.

## Conclusion

Our data show that the use of the AVRC improves the quality of management of critically buried avalanche victims in CA. The AVRC enables the identification and appropriate treatment of patients with hypothermic CA. Inadequate management and incomplete documentation was mainly found in victims with a long burial duration. This category of patients should be the focus of quality improvement efforts. The results of this study encourage generalisation of the use of the AVRC by rescue services responding to avalanche accidents and should facilitate its implementation.

## Electronic supplementary material

Below is the link to the electronic supplementary material.

Additional file 1: Latest version of the AVRC during the study period. Kottmann A, Blancher M, Pasquier M, Brugger H: Avalanche Victim Resuscitation Checklist adaption to the 2015 ERC Resuscitation guidelines. *Resuscitation* 2017, 113:e3-e4

Additional file 2: Pre-hospital medical record (PHMR) from the Swiss HEMS Rega – Swiss Air Ambulance (2024)

## Data Availability

No datasets were generated or analysed during the current study.

## Abbreviations

AVRC: Avalanche Victim Resuscitation Checklist
CA: Cardiac arrest
ECG: Electrocardiogram
ECLS: Extracorporeal life support
HEMS: Helicopter emergency medical service
HOPE score: Hypothermia outcome prediction after ECLS score
ICAR MEDCOM: International Commission for Mountain Emergency Medicine
PHMR: Pre-hospital medical record
