# Supplementary material for: The impact of a dedicated checklist on the quality of onsite management of critically buried avalanche victims in cardiac arrest in a Swiss helicopter emergency medical service
Source: Scand J Trauma Resusc Emerg Med. 2024 Dec 3;32:124. doi: 10.1186/s13049-024-01300-3 (PMC11613841; doi:10.1186/s13049-024-01300-3)

**Additional file 1: Latest version of the AVRC.** Kottmann A, Blancher M, Pasquier M, Brugger H: **Avalanche Victim Resuscitation Checklist adaption to the 2015 ERC Resuscitation guidelines**. *Resuscitation* 2017, **113**:e3-e4.


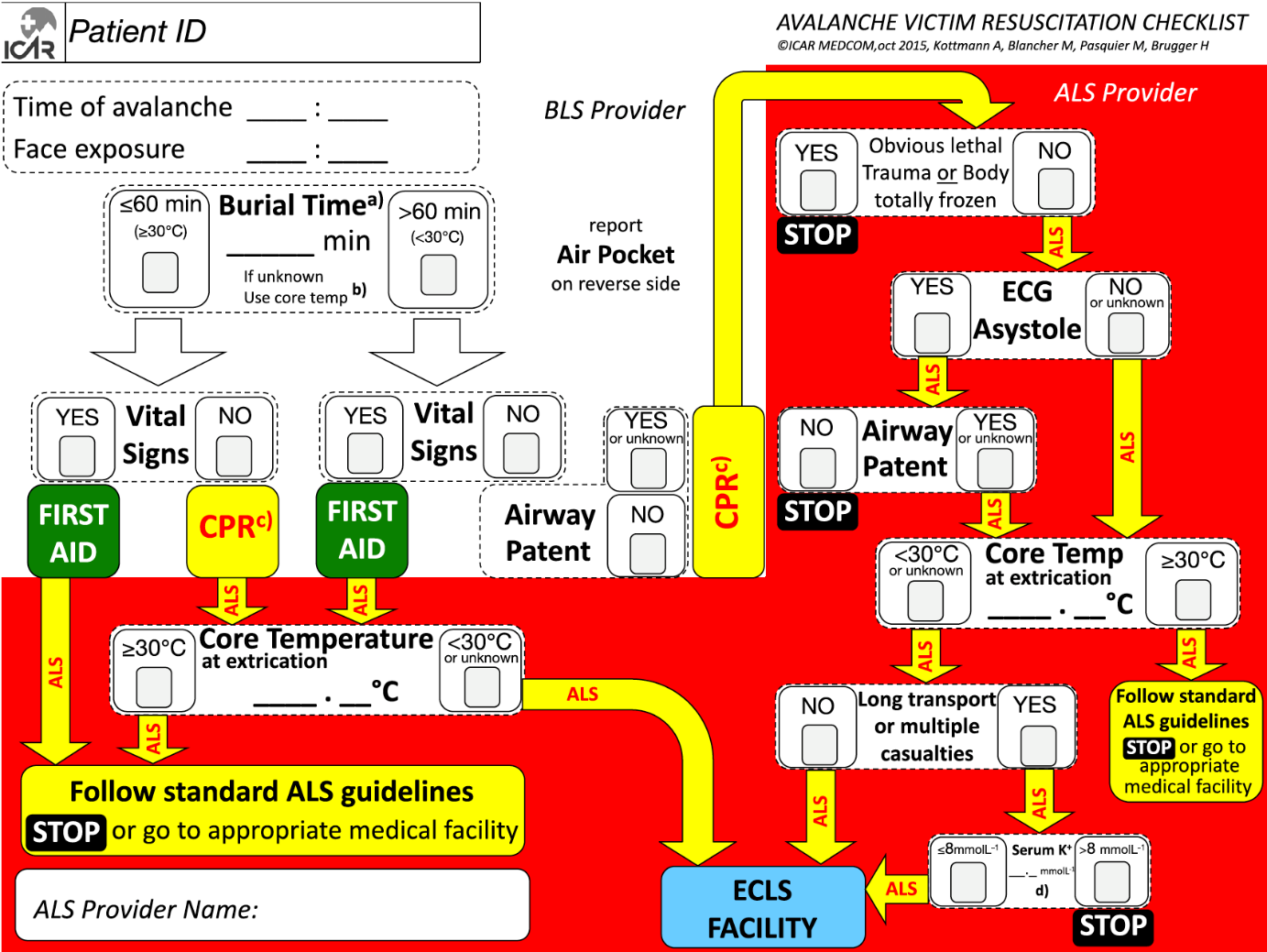

Supplement: Supplementary file 1 — Additional file 1: Latest version of the AVRC during the study period. Kottmann A, Blancher M, Pasquier M, Brugger H: Avalanche Victim Resuscitation Checklist adaption to the 2015 ERC Resuscitation guidelines. Resuscitation 2017, 113:e3-e4 [file 13049_2024_1300_MOESM1_ESM.docx]
