# Supplementary figures and images for: The impact of a dedicated checklist on the quality of onsite management of critically buried avalanche victims in cardiac arrest in a Swiss helicopter emergency medical service
Source: Scand J Trauma Resusc Emerg Med. 2024 Dec 3;32:124. doi: 10.1186/s13049-024-01300-3 (PMC11613841; doi:10.1186/s13049-024-01300-3)

**Additional file 2: Pre-hospital medical record (PHMR) from the Swiss HEMS Rega – Swiss Air Ambulance (2024)**


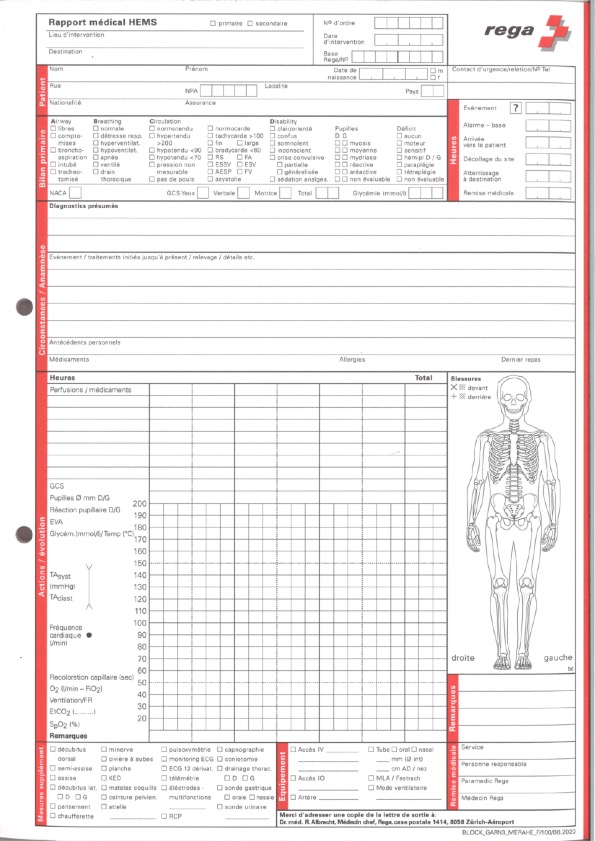

Supplement: Supplementary file 2 — Additional file 2: Pre-hospital medical record (PHMR) from the Swiss HEMS Rega – Swiss Air Ambulance (2024) [file 13049_2024_1300_MOESM2_ESM.docx]
